# Effectiveness of a training program for a patient with non-cardiac chest pain that combines intervention to improve quality of life, psychological state, and functional capacity: a case report

**DOI:** 10.1186/s13030-023-00283-4

**Published:** 2023-07-26

**Authors:** Irem Huzmeli, Aysel Yildiz Ozer

**Affiliations:** 1 Department of Physiotherapy and Rehabilitation, Faculty of Health Science, Hatay Mustafa Kemal University, Hatay, Turkey; 2 Department of Physiotherapy and Rehabilitation, Faculty of Health Sciences, Marmara University, Istanbul, Turkey

**Keywords:** Noncardiac chest pain, Quality of life, Functional capacity, Depression, Case report

## Abstract

**Background:**

Noncardiac chest pain (NCCP) is persistent angina-like chest pain without cardiac origin that affects the patient’s health related quality of life (HrQoL), although it does not affect mortality. The effect of a comprehensive training program for NCCP focused on quality of life, psychological state, peripheral muscle strength, physical activity, and quality of life has not been previously established or published. Therefore, this study aimed to show the effectiveness of our combined training program that focuses on functional capacity, physical activity, pulmonary function, respiratory and peripheral muscle strength, dyspnea, fatigue, anxiety, and depression perception in NCCP patients with dyspnea.

**Case presentation:**

A 38-year-old man with shortness of breath and NCCP was referred to to us for cardiopulmonary rehabilitation. Respiratory muscle strength (mouth pressure device), functional capacity (6 min walking test, 6-MWT), peripheral muscle strength (dynamometer), pulmonary function (spirometry), fatigue (fatigue severity scale), shortness of breath (MMRC, Modified Medical Council Research, Modified Borg Scale-MBS), physical activity (International Physical Activity Questionnaire, IPAQ), health related quality of life (SF-36, Short Form-36), and depression and anxiety (Hospital Depression and Anxiety scale, HADs) were assessed. Aerobic training combined with inspiratory muscle training (loading 30% maximal inspiratory pressure (MIP)) was administered at least 5 days/week for 6 weeks. Functional capacity, physical activity, pulmonary function, and respiratory and peripheral muscle strength improved, and dyspnea, fatigue, anxiety, and depression perception were decreased after the management.

**Conclusions:**

This combined training program was effective for patients with NCCP and shortness of breath. Future studies should be conducted to find the most effective biopsychosocial training protocol for NCCP patients.

**Supplementary Information:**

The online version contains supplementary material available at 10.1186/s13030-023-00283-4.

## Details of the clinical case

Noncardiac chest pain (NCCP) is a pain that is not of cardiac origin that affects the person’s life for an unknown reason. Although the history and characteristics of NCCP cannot be reliably distinguished from cardiac and esophageal chest pain, it is widespread in the general population. It presents a significant burden to providers across clinical settings. The literature reports chest wall causes in 36%, gastrointestinal causes in 19%, cardiac causes in 16%, psychiatric causes in 8–50%, and pulmonary causes in 5% of patients with NCCP [1]. The broad distinguishing diagnosis for chest pain and the possible mortal results of missing an extremely life-alarming medical situation add complexity to clinical decision-making [2–3].

We recently received a referral to our cardiac rehabilitation program for the management of a 38-year-old man who had been diagnosed with NCCP and shortness of breath. His medical history was not remarkable other than that his symptoms were reported to have begun two years ago after an uncomplicated cholecystectomy. His body mass index could be considered high (24.7 kg/m^2^).

Testing was done for acute coronary syndrome, aortic dissection, pulmonary thromboembolism, and pericardial tamponade as possible causes of the chest pain. To test for the presence and/or severity of chronic ischemic heart disease or pericardial disease, an electrocardiogram (ECG) was done during an exercise test. The possibility of cardiac angina was investigated. Because NCCP with shortness of breath differs from cardiac angina in that the patient is usually younger, does not have typical angina symptoms, has a normal resting ECG, and has a higher level of anxiety than normal [4], clinical diagnosis was determined as NCCP.

This case raises several interesting issues of importance of physical therapists in the assessment, prescription of intervention, and disease progression of patients with NCCP. We prescribed a program of aerobic exercise and inspiratory muscle training (at least five times a week for six weeks). However, we recognize that the presentation of these patients is complex. Thus, they warrant detailed assessment and comprehensive interventions, not unlike those patients with chest pain and shortness of breath related to underlying heart disease. We share these reflections with the health professions community, hoping that they will help identify and guide the management of such patients. In addition, our observations may help develop much-needed research programs related to this interesting patient cohort.

### Clinical report

The patient had a history of cholecystectomy, never smoked, drank alcohol, and had no regular exercise habits. He had a history of dyspnea/shortness of breath. Activities that trigger exertional dyspnea include climbing stairs, going uphill, and walking. Dyspnea on MBS was three at rest and 5 with activity. He had visited a general surgery clinic for acute cholecystectomy on September 2, 2017. He was not recommended any new drugs during the program, but was temporarily started on 1 × 1 Dideral (cardiac stress preventative) medication from the cardiology clinic. Dideral 40 mg (1 × 1) is effective in conditions such as hypertension, heart angina, controlling heart rate due to anxiety, and depression.

Exercise stress myocardial perfusion scintigraphy report:

Target HR: 183 beats/min Recovery HR:176 (%98) Angina: (-) Reason for stopping: fatigue. Left ventricular walls were within normal limits, and the Left Ventricular ejection fraction was 65%.

Laboratory Findings: Glucose: 100 mg/dl, Hemoglobin: 16.8 g/dL, Cholesterol: 93 mg/dl, Triglyceride: 178 mg/dL, HDL cholesterol: 32 mg/dL, LDL cholesterol:25 mg/dL Hemotocrit:47.5% Blood Urea Nitrogen:20 mg/dL, Creatinine: 0.84 mg/dL.

### Assessments

The Patient gave informed consent for participation in the study and publication of the report. All assessments were performed at baseline and at the end of 6 weeks. Lung volume was measured with a pulmonary function device (MiniSpir Spirobank 2 Advanced, England), respiratory muscles with a portable mouth pressure measuring device (MicroRPM, CareFusion, UK), and peripheral muscle strength with a portable dynamometer. A change of 100 ml for respiratory functions is considered the minimal clinically important difference (MCID) value [5]. While the MCID value for MIP was set at 11 cmH_2_O, according to literature [6]; the change in muscle strength of the knee extensors was not calculated, and a 17 N chance was taken as a reference from the Knols et al’ study [7]. Functional exercise capacity was evaluated with the 6-MWT. Six minutes of walking work (6MWORK, kg·m) was calculated by multiplying the patient’s body weight (kg) by the 6-minute walking distance (m) [8]. According to the literature, 6MWT MCIDs of 14.0 to 30.5 m after the ROC analysis were reported in randomised controlled trials. Hence, changes in 6MWT distance that are greater than 30.5 m can be considered clinically significant and to meet a realistic threshold for a true change in 6MWT distance [9]. Myocardial oxygen consumption was calculated with the following formula: [heart rate X systolic blood pressure X 10^− 2^ (beats/min. mmHg) [10]. To evaluate dyspnea during routine activities, the MMRC was employed. Scores for dyspnea ranged from 0 (no symptoms during vigorous exercise) to 4. (Dyspnea during all activities of daily living). The fatigue level was assessed by the Fatigue Severity Scale (FSS). A minimum score of 9 is permissible for this scale, whereas a score of 36 or above indicates a high level of fatigue [11]. The Health related Quality of life (HrQoL) was evaluated via the SF-36. To calculate the total physical and mental health components, subscales were used. The SF-36 sub-parameter scores range from 0 to 100. High scores indicate a high degree of life quality [12]. The International Physical Activity Questionnaire - Short Form (IPAQ) was used to calculate the physical activity level. A physical activity level of < 600 MET-min/week is defined as physically inactive, while a physical activity level of > 3000 MET-min/week is considered sufficient [13]. Depression and anxiety levels were evaluated with the Hospital Anxiety and Depression Scale (HADs) [14, 15]. The MCID value for HADs is reported as 5.7 in patients with orthopedic problems [16], 1.7 in patients with cardiovascular disease [17], and 2 in patients with bronchiectasis and symptoms of mood disorder [18].

### Therapeutic interventions

The patient was trained with a combined training program for at least 5 days/per week and was followed up for six weeks.

The program contains the following:

1. Inspiratory muscle training
  Type: Inspiratory endurance training with POWERbreathe LR Classic.
  Intensity: 30% of MIP (cmH2O, level 3) 30 Breaths/cycle.
  Duration: 15 min/session six weeks.
  Frequency: 2 times/day, five days/week.
2. Aerobic exercise training
  Type: Brisk walking outdoors.
  Training: 5 min warm-up, 35 min load, 5 min cooldown.
  Intensity: 65% of Maximal Heart Rate (118 beats/min).
  Duration: 45 min/day/6 week.
  Frequency: 1 time/day, 5–7 days/week.

### Follow up and outcomes

The patient’s MMRC scale score was “1” initially and changed to 0 after the program. There was an improvement in pulmonary function, but the difference was not clinically meaningful (the lung function criterion of an MCID of 100 ml), and upper, lower extremity, and respiratory muscle strength were increased after the program [5]. Because the change in muscle strength of the knee extensors was not calculated for NCPP patients, the minimal clinical significance value determined by Knols et al. for a different disease group was taken into consideration, and the minimal clinical significance value was accepted as 17 N [7]. MCIDs were 6.5 kg for grip strength and 19.5% for percentage grip strength. The MCID was not less than the minimum detectable change for grip strength (also 6.5 kg) [19]. The patient’s fatigue perception was low, as indicated by the score (31) obtained from the scale, lower than 36, the cutoff point. The total point of FSS (from 31 to 14) and HADs (from 18 to 11) were decreased after the program. According to Lemay et al., 2019, the Minimal Clinically Important Difference (MCID) for the HADs in patients with cardiovascular disease is 1.7 points [17]. A MCID was found in the HADs total (pre:18, post: 11, MCID:7), depression (pre:8, post: 5, MCID:3), and anxiety (pre:10, post: 6, MCID:4) subscale scores after the combined program for this patient with NCCP. Bohannon and Crouch, 2017 found that a change of 14.0 to 30.5 m may be clinically important for patients with cardiovascular disease. Our NCCP patient’s MCID was 113 m (from 517 to 630 m) [9]. The patient became minimally active, and his general health and social participation improved. Functional capacity increased from 517 to 630 m, and the difference (113 m) was clinically significant (MCID: 30.5 m) [9] (Table 1).

**Table 1:** Pulmonary function, peripheral and respiratory muscle strength and functional capacity results

	Pre-intervention			Post-intervention		
*Pulmonary functions*	L	%		L	%	
FVC	4.44	85		4.62	89	
FEV1	3.72	86		3.78	87	
FEV1/VC (%)		-			85.1	
FEF 25–75%	3.86	79		3.78	78	
PEF	7.66	78		8.24	83	
*Peripheric muscle strengths*	R	L		R	L	
Knee extensors (N)	220	79.2		242	85	
Hip Flexors (N)	209	149		220	171	
Shoulder Abductors (N)	101	96.8		125	101	
Elbow Flexors (N)	132	100		136	118	
Hand Grip strength (kg-F)	118	104		125	110	
*Respiratory muscle strengths*						
MIP (cmH2O)	81			115		
MIP %	65.29			92.69		
MEP (cmH2O)	61			71		
MEP %	26			30		
*IPAQ* (MET-min/week)						
Vigorous activity	0			800		
Moderate activity	0			120		
Walking	346.5			1039.5		
Total	886.5			2586.5		
Sitting	450			630		
Physical activity categories	Inactive			Minimal active		
**FSS score**	31			14		
***SF-36-***Physical functioning	95			95		
***SF-36-***RL-PH	0			100		
***SF-36-***RL-EP	0			100		
***SF-36-***Energy/fatigue	25			30		
***SF-36-***Emotional well-being	44			44		
***SF-36-***Social functioning	12.5			62.5		
***SF-36-***Pain	30			55		
***SF-36-***General health	70			80		
***HADs*** Total Score	18			11		
***HADs*** Categorization	Probable			Possible		
***HADs depression score***	8			5		
***HADs anxiety score***	10			6		
**Functional capacity results**	Rest	After	1stmin recovery	Rest	After	1stmin recovery
Heart rate (beat/min)	84	98	88	79	99	89
SpO2 (%)	97	97	98	98	98	99
Blood pressure (mmHg)	130/90	130/80	110/70	100/70	149/70	125/60
Respiratory rate (breathe/min)	14	20	15	18	24	20
Dyspnea (MBS, 0–10)	1	5	4	0	0	0
Fatigue (0–10)	3	5	4	0	2	0
Lower extremity fatigue, 0–10)	0	2	0	0	0	0
MOC (beats/min.mmHgx10^− 2^)	109.2	127.4	96.8	79	147.51	111.25
6MWT (m)	517			630		
6MWT (%)	66			81		
6MWORK( kg·m)	41.440			50.400		

HADs: Hospital Depression and Anxiety Scale, FSS: Fatigue Severity Scale, MBS: Modified Borg Scale, MOC: Myocardial oxygen consumption, RL-EP: Role limitations due to emotional problems, RL-PH: Role limitations due to physical health, 6MWT: 6-min Walking Test, R: Right, L: Left, N: Newton

## Discussion

With this comprehensive training program, an improvement in respiratory and peripheral muscle strength, HrQoL, physical activity level, functional capacity, and a decrease in the perception of fatigue, anxiety, depression, and dyspnea were observed after treatment of this patient with unexplained chest pain and shortness of breath. NCCP is persistent angina-like pain that cannot be attributed to coronary heart disease based on standard diagnostic testing. Its origin may be from problems of chest wall, gastrointestinal system, cardiac, psychiatric, or pulmonary causes. Psychological components of disease have been highlighted in therapeutic approaches. Moreover, NCCP and the feeling of not being able to breathe can cause severe physical inactivity in patients, resulting in increased fatigue and decreased functional capacity, which adversely affects HrQoL [4, 20]. Therefore, as in this case of NCCP, we believe in the benefit of additional treatment programs such as regular combined comprehensive exercise programs in addition to medical treatment. In our program, considering the psychological factors related to NCCP, we called the patient once a week and checked his app chart weekly via WhatsApp. We confirmed for NCCP the effectiveness of device-guided respiratory muscle training and aerobic training and the positive effects of the combined training, as has been seen for other diseases. Inspiratory muscle training combined with cardiac rehabilitation has been recommended in studies to significantly increase functional capacity.

A study in which three different interventions were applied to post-MI patients found that cardiac rehabilitation combined with inspiratory muscle training was the most effective intervention [21]. In a previous study, in heart failure patients who received ARIS (aerobic + resistance training + respiratory muscle training), aerobic/respiratory muscle training, aerobic / resistance training, and aerobic training were applied three times a week for 180 min for a total of 12 weeks. ARIS was found to show the best improvement, especially in aerobic capacity, left ventricle dimensions, and secondary factors measures [22]. A 6-week Inspiratory Muscle Training Combined with exercise was effective in terms of functional capacity, peripheral and inspiratory muscle strength, and lung volumes in patients with lung cancer who underwent video-assisted thoracoscopic surgery [23]. A recent systematic review and meta-analysis on combined aerobic and resistance training after coronary artery disease stated that moderate intensity, 2–3 days a week, 50 min (aerobic training)/day, three sets of 10–12 repetitions, and seven exercises for 14 weeks was effective in improving peak oxygen uptake and muscle strength [24]. Similar to previous studies, our study showed improvement after six weeks of the combined training program in functional capacity (MCID: 30.5 m), pulmonary function, dyspnea perception (MMRC:0), quality of life, physical activity (from 886.5 to 2586.5 (MET-min/week), fatigue (from 31 to 14), anxiety (from 18 to 11) perception, and peripheral and respiratory muscle strength (MIP from 81 to 115 cmH2O & MEP 61 to 71 cmH2O). Our combined training applied for six weeks positively increased myocardial oxygen consumption and 6 min work. This resulted in positive efficiency of the left ventricle. Therefore, the patient may have decreased chest pain and feeling of shortness of breath after the combined rehabilitation program.

Although there is no specific research on the clinical effects of unexplained dyspnea, there is no suspicion that persistent respiratory distress represents a significant threat of morbidity.

The contribution of inspiratory muscle weakness (i.e., without accompanying cardiorespiratory or neuromuscular illness) to dyspnea and the exercise response pattern is not fully defined. In fact, the number of unique studies defining physiological and sensory replies is currently insufficient. In particular, dyspnea during exercise (exertional dyspnea) has been attributed to inspiratory muscle weakness. When subjects with inspiratory muscle weakness showing symptoms of overexertion (shortness of breath and leg discomfort) were compared with controls, a higher metabolic and ventilation demand for a given work rate in serial measurements of lung volumes and symptomatic perception during submaximal exercise was observed. Decreased inspiratory capacity associated with an excessive tachypnea breathing pattern and preserved oxygen gas exchange indicates inspiratory muscle weakness that contributes to effort intolerance and dyspnea [25]. The physical activity level of our patient who received the combined training changed from sedentary to minimally active, and the level of physical activity increased. Castonguay et al. found that being physically active was associated with being less likely to suffer from an NCCP-related disability within six months of an emergency room visit [26]. Mourad et al. stated that NCCP was associated with psychological distress and decreased HrQoL, and patients with NCCP may benefit from additional therapies aimed at reducing psychological distress and improving HrQoL [27]. Indeed, depression and anxiety are common in patients with chronic disease, as well as in those with acute coronary syndrome (ACS) or other major cardiovascular events. Furthermore, both depression and anxiety have been independently associated with negative cardiac outcomes in patients with acute cardiac events and, indeed, across the spectrum of cardiac disease, especially angina [28]. Anxiety and depression have been associated with adverse societal and individual correlates, including higher health care costs and an increased risk for physical comorbidities, such as cardiovascular illnesses [29, 30]. Moreover, they have been linked to a reduced quality of life (QoL) in numerous cross-sectional as well as longitudinal studies in which they significantly predicted QoL outcomes [31]. Quality of life was significantly more impaired in panic disorder patients than in chest pain patients without panic disorders [32]. This result shows the effect of psychological status on quality of life.

Despite their frequency, these psychiatric syndromes are often undetected in the illness period and may persist for months to years, with the effect of the patient taking other symptoms more seriously. This negative process significantly negatively affects the individual’s physical activity participation and quality of life over time. The result will be the development of a vicious cycle of pain-psychiatric problems and inactivity-impairment in quality of life. We believe that this vicious cycle can be broken with our combined training program. Indeed, during our program, no side effects were observed in the patient with NCCP. We could not evaluate chest pain with more objective methods, and this was one of our limitations. On the other hand, at the end of the program, quality of life and functional capacity increased, while dyspnea, the perception of fatigue, depression, and anxiety decreased. In the process of breaking this vicious cycle; support and participation were increased with motivational interviewing; mental and physical gains were supported with physical activity and exercises; pain was reduced by increasing well-being mood and social interaction; and an improvement in the quality of life was achieved. Our results are evidenced that this combined training can safely be applied to patients with NCCP to increase the physical activity level, reduce psychological distress, and improve HrQoL.

Lastly, physical exercise has been reported to modulate major central nervous system neurotransmitters such as norepinephrine (noradrenalin), which is associated with alertness, dopamine, which is involved in reward-motivated behavior, and serotonin, which is widely thought to contribute to feelings of well-being and happiness. Although the inhibitory effect of exercise on depression, anxiety, and stress levels by modulating central nervous system neurotransmitters is emphasized in the literature [33] we did not evaluate these parameters in this case. However, future randomized controlled studies will be done examine the effects of exercise on physiological mechanisms in this patient group.

Respiratory muscle fatigue or weakness has been shown to be associated with exercise restriction in healthy individuals and patients with heart failure [34, 35]. Although it was determined by objective data that respiratory muscles were strengthened and lung function was better, the “SF-36 Physical Functioning” score did not change after the program. We used common scales to evaluate the quality of life and some subjective parameters. One of the major disadvantages of this situation is that the results obtained are based on self-report [36]. Long-term pain experience can seriously affect the individual psychosocially. This situation can also lead to a deterioration in body awareness. Although the patient’s compliance with the program was good and objective test results showed improvement, the patient may not be aware of this improvement. Considering the psychosocial dimension of NCCP, we think that providing body awareness training to these patients can improve their compliance with the treatment process and symptom awareness. This program will help patients become aware of improvements in symptoms that they are not aware of.

## Summary

At the end of the comprehensive combined exercise training, the patient had improved his symptoms on quality of life, functional capacity, dyspnea, fatigue perception, and psychological status. We have yet to conduct a follow-up to see if these improvements persist. Entry into our cardiorespiratory rehabilitation program was an essential gateway for this patient to establish his overall health and well-being and functional capacity. A comprehensive health and lifestyle framework that provides for the cardiorespiratory health of all patients would be helpful for achieving even more significant potential benefits than were observed in this case.

## Electronic supplementary material

Below is the link to the electronic supplementary material.

Supplementary Material 1

## Data Availability

The datasets used and/or analyzed during the current study are available from the corresponding author (IH) upon reasonable request. Because it contains personal information about the patient.

## Abbreviations

6-MWT: 6 min walking test
HADs: Hospital Anxiety and Depression Scale
IPAQ: International physical activity questionnaire
MBS: Modified Borg Scale
MIP: Maximal inspiratory muscle
MMRC: Modified medical council research
NCCP: Non cardiac chest pain
HrQoL: Health related quality of life
