# Supplementary material for: Effectiveness of a training program for a patient with non-cardiac chest pain that combines intervention to improve quality of life, psychological state, and functional capacity: a case report
Source: Biopsychosoc Med. 2023 Jul 26;17:27. doi: 10.1186/s13030-023-00283-4 (PMC10369802; doi:10.1186/s13030-023-00283-4)
Supplement: Supplementary file 1 — Supplementary Material 1 [file 13030_2023_283_MOESM1_ESM.docx]

**Dear Editor,**

We appreciate you giving us the chance to improve our content. We fixed and updated the text to reflect the opinions of the helpful reviewers and their constructive suggestions. We would also want to thank the reviewers for their efforts. Furthermore, we have marked in yellow all of the altered sections of the paper.

Best regards.

Reviewer reports

**Reviewer #1:** Thank you for your efforts.

Thank you for your valuable contribution to our article.

**Reviewer #3:** I have made numerous corrections that I hope you find helpful.

We reviewed the article in its entirety and finalized our modifications based on your comments. Thank you for your suggestions and important contributions.
